# Politische Kindermedizin: Für eine bessere Zukunft aller Kinder

**DOI:** 10.1007/s00112-021-01140-w

**Published:** 2021-03-15

**Authors:** C. Popow, R. Püspök, E. Tatzer, S. Gobara, O. Jürgenssen

**Affiliations:** 1 Univ. Klinik für Kinder- und Jugendpsychiatrie, Medizinische Universität Wien, Währinger Gürtel 18–20, 1090 Wien, Österreich; 2 Landesklinikum Mauer, Mauer bei Amstetten, Österreich

**Keywords:** Kindeswohl, Medizinische Versorgung, Lobbying, Verbesserungen, Österreich, Child welfare, Pediatric care, Lobbying, Improvements, Austria

## Abstract

Die „Politische Kindermedizin“ ist ein Projekt engagierter KindermedizinerInnen, die Missstände und Fehlentwicklungen in der Kindermedizin analysieren, bewerten und aufzeigen, Lösungsvorschläge erarbeiten und diese an die Politik herantragen. Ziel war und ist es, im Sinne von „patient advocacy“ eine Verbesserung der pädiatrischen Versorgung von Kindern und Jugendlichen zu erreichen. Geschichte und Anliegen der Initiative „Politische Kindermedizin“ werden von 1997 bis heute dargestellt.

## Abstract (en)

Advocacy for pediatric care (“Politische Kindermedizin”) is an Austrian association of engaged pediatricians, aiming at identifying, analyzing and evaluating pressing issues of pediatric care and to suggest improvements to the responsible politicians. The aim was and is to achieve an improvement of pediatric care for children and adolescents in the sense of patient advocacy. The history and concerns of the initiative “Politische Kindermedizin” from 1997 until the present are presented.

## Geschichte

Die „Politische Kindermedizin“ begann in den 1990er-Jahren als gemeinsames Projekt einer Gruppe von Freunden, die sich durch ihre Tätigkeit an der Universitätskinderklinik in Wien kannten. Ihr Anliegen war, Unzufriedenheit mit der medizinischen Versorgung von Kindern und Jugendlichen nicht zu bejammern, sondern durch faktenbasierte Analyse Vorschläge zu einer Systemverbesserung zu erstellen, öffentlich bewusst zu machen und ihre Umsetzung auf politischem Weg zu erreichen. Eines der wichtigsten Gründungsmitglieder, Franz Waldhauser, musste seine Koautorenschaft bei diesem Beitrag zurückziehen, da er in einem Gerichtsverfahren vertraglich dazu verpflichtet wurde, keine weitere Kritik am System zu üben.

Die erste Initiative dieser Gruppe war eine Bestandsaufnahme, das Buch *Weggelegt. Kinder ohne Medizin* [11]: In 15 Kapiteln wurde die Kritik am System von verschiedenen ExpertInnen engagiert dargestellt. *Weggelegt* erregte Aufsehen v. a. durch ein Kapitel über die Ergebnisse der Kinderherzchirurgie und die im internationalen Vergleich geringere Zahl von Lebertransplantationen in Österreich. Das System reagierte massiv gekränkt mit einer zivilrechtlichen Klage der Gemeinde Wien und einem Disziplinarverfahren der Medizinischen Universität Wien gegen den Erstautor sowie disziplinären Drohungen gegen die Mitautoren des Kapitels. Der Rechtsstreit endete mit einem Vergleich^1^, das Disziplinarverfahren mit einem Freispruch.

Trotz schwieriger Anfänge tritt die PKM weiterhin für eine gute und gerechte Versorgung aller Kinder ein

Die Konsequenz war allerdings eine Systemreform: Das Gesundheitsministerium richtete eine international besetzte Kommission ein; die Kinderherzchirurgie wurde in Österreich neu organisiert. Die Behandlungsergebnisse, die bis dahin auch nicht veröffentlicht worden waren, liegen mittlerweile im europäischen Spitzenfeld [12, 13]. Im Weiteren initiierte das Gesundheitsministerium eine Kinder- und Jugendgesundheitsstrategie [14], wobei auch Autoren von *Weggelegt* in die Konzeption eingebunden wurden. Auch die Österreichische Gesellschaft für Kinder- und Jugendheilkunde (ÖGKJ) hat große Teile des Inhalts von *Weggelegt* zu ihren Anliegen gemacht.

Die nächsten Schritte waren eine Verbreiterung der Basis durch öffentliche Diskussionsrunden, die Einrichtung einer Homepage (www.polkm.org) und die Gründung mehrerer Arbeitsgruppen:

- *„Kind – arm – krank“* (Ernst Tatzer), mit sozialpädiatrischen Anliegen, insbesondere zur Verbesserung der therapeutischen Versorgung von Kindern mit besonderen Bedürfnissen,
- *„Spezialisierung in der Kindermedizin“*, später „Schwerpunktsetzung in der Pädiatrie“ (Franz Waldhauser), die sich mit der Struktur der stationären pädiatrischen Versorgung, insbesondere der tertiären Versorgung, befasst,
- *„Selbsthilfegruppen“* (Irene Promussas), die dann in „Lobby4Kids“ [15], einen Dachverein für Selbsthilfegruppen überging.

Weitere Schritte waren die Herausgabe von vierteljährlichen Newslettern (Redaktion Rudi Püspök; [16]) sowie die Organisation von Jahrestagungen, die aus geografischen und organisatorischen Gründen vorwiegend in Salzburg stattfanden (Organisation Leonhard Thun-Hohenstein). Bereits die erste Tagung zum Thema „Werte vs. Ökonomie“, im Oktober 2007, war ein großer Erfolg, obwohl den Mitarbeitern der Universitätskliniken in Wien und Graz von einer Teilnahme „dringend abgeraten“ wurde. Bei dieser und allen weiteren Tagungen (Tab. 1) wurde auch eine am Ende der Tagung^2^ von den TeilnehmerInnen erarbeitete Resolution [17] erstellt. Schriftliche Fassungen der Vorträge wurden als Buch bzw. Sonderheft im Springer Medizin Verlag veröffentlicht und an ausgewählte EntscheidungsträgerInnen des österreichischen Gesundheitssystems versandt. Eine frühe Darstellung der Aktivitäten der Initiative „Politische Kindermedizin“ findet sich in einem Themenheft der *Monatsschrift für Kinderheilkunde* des Jahres 2010 [3, 10].

**Table 1:** 

Tagung	Datum	Themen^a^
1	19.–20.10.2007	Werte vs. Ökonomie
2	24.–25.10.2008	Chronisch krank – chronisch unterversorgt?
3	16.–17.10.2009	Kind und Recht
4	12.–13.11.2010	Kinder im besten Gesundheitssystem der Welt?
5	14.–15.10.2011	Kind, Medizin, Medien und Politik
6	19.–20.10.2012	Probleme der kinder- und jugendmedizinischen Primärversorgung in Österreich
7	15.–16.11.2013	Partizipation in der Kinder- und Jugendmedizin – von der Versorgung zur Teilhabe
8	06.–07.11.2014	Kompetenzzentren und Versorgungsnetzwerke für Kinder und Jugendliche mit seltenen, komplexen und diagnostisch/therapeutisch aufwendigen Erkrankungen
9	16.–17.10.2015	Lost in Transition – Wenn aus Kindern Erwachsene werden
10	11.–12.11.2016	Im Netz geborgen? Netzwerke und ihre Wirkung
11	10.–11.11.2017	Welcome? Medizinische Versorgung von Flüchtlingskindern
12	09.–10.11.2018	Medizinisch-therapeutische Versorgung von Kindern und Jugendlichen: Wer hat die Verantwortung? Wer nimmt sie wahr?
13	08.–09.11.2019	Seltene Erkrankungen. Gemeinsam mit dem Forum „Seltene Erkrankungen“ und „Pro Rare Austria“
14	Verschoben auf 11.–12.11.2021	Bildung und Gesundheit

^a^Vgl. die am Ende unserer Homepage, http://www.polkm.org, aufgeführten Proceedings der Jahrestagungen

In der Folge wurden der Aktualität der Themen entsprechend weitere Arbeitsgruppen gebildet:

- *Primärversorgung* (Ernst Tatzer, später Rudi Püspök),
- *Flüchtlingsmedizin* (Nicole Grois),
- *Transition* (Christian Popow, später Sonja Gobara).

Die Arbeitsgruppe „Kostenfreie Therapien“ (Rudi Püspök, später Irmgard Himmelbauer) setzte die Bemühungen um notwendige (für die Eltern) kostenfreie funktionelle Therapien und Psychotherapie [18] fort. Dabei ist mit „kostenfrei“ die Umsetzung der im Allgemeinen Sozialversicherungsgesetz verankerten Verpflichtung zur Finanzierung notwendiger Therapien durch die Sozialversicherungen gemeint. Diese Befreiung von für die meisten Familien nichtleistbaren Selbstkosten bei Inanspruchnahme privater Leistungserbringer ist deshalb notwendig, weil das Angebot an krankenkassenfinanzierten Therapieplätzen viel zu gering ist [19]. Gespräche mit den Krankenkassen, dem Hauptverband der Sozialversicherungsträger und dem Gesundheitsministerium trafen immer auf Wohlwollen, gleichzeitig aber auch auf geringe Bereitschaft, das „System“ (das den Sozialversicherungsträgern durch Nichtleistung Ersparnisse in Millionenhöhe auf Kosten der betroffenen Kinder und deren Familien beschert), zu ändern. Wichtige Bausteine waren:

- eine *Bedarfserhebung* durch Rudi Püspök [7] anhand der in Deutschland erbrachten Leistungen und des Vorbilds des Vorarlberger Modells (aks Gesundheit Vorarlberg [20]),
- die *Erstellung und Erprobung eines Verordnungskatalogs* für funktionelle Therapien (Sonja Gobara, Othmar Fohler, Irmgard Himmelbauer, Klaus Kranewitter und Rudi Püspök, [21]) und
- *Kontakte* zum Hauptverband der Sozialversicherungsträger und zum Gesundheitsministerium

Seit 2013 ist die Plattform „Politische Kindermedizin“ als gemeinnütziger Verein organisiert. War die „Politische Kindermedizin“ zunächst angefeindet (s. „Weggelegt“), so hat sie sich in den letzten Jahren zu einem verlässlichen Partner und Ideengeber entwickelt, wozu auch die Kontakte zur Österreichischen Gesellschaft für Kinder- und Jugendheilkunde (Daniela Karall, Reinhold Kerbl, Willy Kaulfersch, Klaus Schmitt, Wolfgang Sperl) und zur Österreichischen Gesellschaft für Kinder- und Jugendpsychiatrie (Leonhard Thun-Hohenstein) sowie zu in- und ausländischen Wissenschaftlern und zur Politik beigetragen haben.

„Politische Kindermedizin“ hat sich zu einem verlässlichen Partner und Ideengeber entwickelt

Leider haben zuletzt die durch die „coronavirus disease 2019“ (COVID-19) bedingten Mobilitäts- und Kontaktbeschränkungen die persönlichen Kontakte und damit die so wichtige Diskussion deutlich eingeschränkt, wozu auch die Verschiebung der 14. Jahrestagung gezählt werden muss. Wir versuchen daher, die zahlreichen „FreundInnen“ der Initiative „Politische Kindermedizin“ mit den 4‑mal jährlich erscheinenden Newslettern [16], den ebenfalls 4‑mal jährlich erscheinenden Newslettern der Arbeitsgruppe „Schwerpunktsetzung in der Pädiatrie“ [22] und auf unserer Homepage zu informieren. Die bisherigen Sprecher bzw. Obleute von „Politische Kindermedizin“ sind in Tab. 2 aufgeführt.

**Table 2:** 

Periode	Sprecher bzw. Obleute
2007–2009	Franz Waldhauser
2009–2012	Reinhold Kerbl
2012–2013	Ernst Tatzer
2013–2017	Sonja Gobara
2017–2019	Ernst Tatzer
2019–2021	Christian Popow

## Anliegen

Die noch immer vordringlichen Anliegen sind:

1. Verbesserung der funktionellen und psychotherapeutischen Versorgung aller Kinder mit Entwicklungs- und psychischen Problemen auf Krankenschein. Hier erscheinen erste Erfolge in einem Gesamtvertrag der ErgotherapeutInnen und Verbesserungen bei den Psychotherapien als Hoffnungsschimmer.
2. Verbesserung der kinder- und jugendpsychiatrischen Versorgung. Diese deckt in den meisten Bereichen nur etwa 50 % der benötigten Kapazität [4]. So fehlen z. B. flächendeckende diagnostische und therapeutische Einrichtungen für Kinder mit Autismus-Spektrum-Erkrankungen.
3. Die Probleme der pädiatrischen Primärversorgung (auch [23]). Der Mangel an Kinder- und JugendfachärztInnen ist bereits eklatant. Immer mehr Krankenkassenordinationen können nicht mehr besetzt werden, was großteils, aber nicht ausschließlich auf die jahrelange finanzielle Unterdotierung der Kassenleistungen zurückzuführen ist [6]. Die „Politische Kindermedizin“ hat hier ein eigenes Zukunftsmodell entwickelt.
4. Diagnostik und Therapie seltener Erkrankungen sollten nicht bei jedem Patienten neu erfunden werden. Daraus ergibt sich die Notwendigkeit einer u. U. überregionalen Spezialisierung und Bündelung von Expertisen [1]. Diese Bestrebungen wurden bereits im europäischen Rahmen realisiert. Österreich hinkt da etwas nach. Die Arbeitsgruppe „Schwerpunkte in der Kindermedizin“ bemüht sich seit Jahren um die Verwirklichung dieses Projekts und setzt sich auch für eine Zentralisierung seltener kinderchirurgischer Operationen in spezialisierten Zentren ein.
  Ein weiteres Anliegen dieser Gruppe, eine patientenzentrierte Finanzierung hochpreisiger Therapien für angeborene seltene Erkrankungen – ist bisher nur teilweise gelungen. Die Mitglieder der Arbeitsgruppe versuchen seit Jahren, eine zentrale Poollösung zu erreichen. Diese Bestrebungen sind bereits recht weit gediehen.
5. Die medizinische Versorgung von geflüchteten Kindern und Jugendlichen sollte standardisiert und besser organisiert werden. Auch auf diesem Gebiet gibt es Verhandlungen auf der Grundlage konkreter Vorschläge [2].
6. Die laufenden Bemühungen um eine Verbesserung der medizinischen Versorgung von Kindern mit besonderen Bedürfnissen finden ihren Niederschlag u. a. in der hoch engagierten Tätigkeit der „Lobby4kids“ [15].
7. Die Bemühungen um eine verbesserte Transition von Jugendlichen mit chronischen Erkrankungen in die Erwachsenenmedizin werden mittlerweile in einem gemeinsamen Arbeitskreis mit der Österreichischen Gesellschaft für Kinder- und Jugendheilkunde (ÖGKJ) organisiert.
8. Zuletzt sind einige Vertreter von „Politische Kindermedizin“, allen voran Franz Waldhauser und Ernst Tatzer, in einer sehr aufwendigen historischen Aufarbeitung den Anschuldigungen nachgegangen, Hans Asperger (der frühere Vorstand der Univ.-Kinderkliniken in Innsbruck bzw. Wien) hätte in der NS-Zeit die „eugenischen“ Tötungen in der Jugendfürsorgeanstalt Am Spiegelgrund unterstützt. Nach ihren Recherchen sind diese Vorwürfe, die in der Forderung nach Umbenennung des Asperger-Syndroms gipfelten, nicht haltbar. Eine wissenschaftliche Auseinandersetzung erschien als Sonderheft der *Monatsschrift für Kinderheilkunde* [5, 8, 9]; die Ergebnisse ihrer Aufarbeitung werden weiterhin in einer internationalen Diskussion vertreten.

## Fazit

- „Politische Kindermedizin“ ist eine Initiative zur Verbesserung der Rahmenbedingungen des Gesundheitssystems für Kinder und Jugendliche – vergleichbar dem englischen Begriff „child advocacy“.
- „Politische Kindermedizin“ ist ein „Minderheitenprogramm“ mit oft langen und vergeblichen Wegen; sie fordert von den Beteiligten viel Durchhaltevermögen und Frustrationstoleranz.
- Der Kontakt zu politisch Verantwortlichen ist für Kinder- und Jugendmediziner nicht alltäglich und muss erst „gelernt“ werden.
- Bei entsprechendem Durchhaltevermögen können wichtige Anliegen umgesetzt werden; in Österreich waren dies u. a. die Kinder- und Jugendlichenrehabilitation, Verbesserungen im Bereich funktioneller Therapien sowie der seltenen Erkrankungen.
- Die nachkommende Generation ist leider nur bedingt für eine derartige (ehrenamtliche) Initiative begeisterungsfähig, sodass diese zu einem großen Teil auf den Schultern pensionierter Kinderärzte lastet.

## Footnotes

1 Franz Waldhauser wurde verpflichtet, für immer zu diesem Thema zu schweigen, dafür hat die Gemeinde Wien ihre anteiligen Gerichts- und Anwaltskosten übernommen.

2 Mit Ausnahme der 13. Jahrestagung.
